# Cooperation and Competition of RNA Secondary Structure and RNA–Protein Interactions in the Regulation of Alternative Splicing

**DOI:** 10.32607/actanaturae.26826

**Published:** 2023

**Authors:** M. A. Vorobeva, D. A. Skvortsov, D. D. Pervouchine

**Affiliations:** M.V. Lomonosov Moscow State University, Moscow, 119192 Russian Federation; Skolkovo Institute of Science and Technology, Moscow, 121205 Russian Federation

**Keywords:** RNA structure, long-range interactions, splicing, RNA-binding proteins, regulation

## Abstract

The regulation of alternative splicing in eukaryotic cells is carried out through the coordinated action of a large number of factors, including RNA-binding proteins and RNA structure. The RNA structure influences alternative splicing by blocking *cis*-regulatory elements, or bringing them closer or farther apart. In combination with RNA-binding proteins, it generates transcript conformations that help to achieve the necessary splicing outcome. However, the binding of regulatory proteins depends on RNA structure and, vice versa, the formation of RNA structure depends on the interaction with regulators. Therefore, RNA structure and RNA-binding proteins are inseparable components of common regulatory mechanisms. This review highlights examples of alternative splicing regulation by RNA-binding proteins, the regulation through local and long-range RNA structures, as well as how these elements work together, cooperate, and compete.

## INTRODUCTION

During maturation, most eukaryotic transcripts undergo splicing, a process in which regions called introns are removed, and the remaining exons are joined to form the mature mRNA [1]. In most cases, splicing is catalyzed by a macromolecular complex called the spliceosome, which consists of small nuclear ribonucleoproteins (snRNPs), which in turn consist of small nuclear RNAs (snRNAs) and the associated proteins [2, 3, 4].

The spliceosome recognizes *cis*-regulatory elements in the pre-mRNA, of which the four main classes are the 5’ splice site (5’ss), the 3’ splice site (3’ss), the polypyrimidine tract (PPT), and the branch point sequence (BPS) [5]. However, processing of identical transcripts can occur differently due to the activation of different splice sites in them or due to their use in different combinations. Thus, many different mRNA isoforms can be found in living cells that are formed due to alternative splicing (AS) of pre-mRNAs transcribed from the same gene.

Several main types of AS events can be distinguished, including cassette exon skipping, the use of an alternative 5’ss or 3’ss, intron retention, or mutually exclusive exon choice [6, 7]. According to the current estimates, at least 95% of human genes containing more than one exon are subject to alternative splicing [8, 9]. The coordinated changes in splicing of multiple pre-mRNAs are an integral part of the regulation of a number of cellular processes [10, 11, 12].

AS is regulated by a combination of RNA–protein, RNA–RNA, and protein–protein interactions that occur between *cis*-regulatory elements and *trans*-acting factors [13, 14]. In addition to the key elements described above (5’ss, 3’ss, PPT, BPS), AS is influenced by additional *cis*-regulatory elements, which can be located both in exons and introns, called exonic and intronic enhancers and silencers of splicing. The interaction of enhancers and silencers with *trans*-acting factors stimulates or suppresses the splice site choice, respectively [15]. The outcome of splicing depends on the coordinated action of multiple enhancers and silencers [16].

In this review, we will briefly provide information about the most studied regulation of AS by RNAbinding proteins, discuss the regulation of AS by RNA secondary structure, and then describe the known facts on the joint action of proteins and RNA structure in the regulation of AS.

## REGULATION OF AS BY RNA-BINDING PROTEINS

More than 1,500 RNA-binding proteins (RBPs) are involved in AS regulation [17]. They can be divided into several classes: heterogeneous nuclear ribonucleoproteins (hnRNP), serine/arginine-rich proteins (SR), and others, such as tissue-specific RNA-binding proteins (e.g., NOVA, neuronal PTB/hnRNP I, RBFOX family, etc.) [6]. Here, we will briefly describe examples related to the RNA structure, while more detailed information on AS regulation by various RBP classes can be found in other reviews [6, 18, 19, 20].

The ubiquitously expressed SR and hnRNP proteins are the best-studied mediators of splice site recognition [21, 22, 23, 24, 25]. SR proteins are involved in both constitutive and alternative splicing, making this RBP family unique compared to other RBPs [22]. SR proteins are generally considered to be positive splicing regulators; they promote exon inclusion by helping to recruit U1 snRNP to the 5’ss and U2 auxiliary factor (U2AF) to the 3’ss through protein–protein interactions during the early stages of spliceosome assembly [21, 26].

SR and hnRNP proteins are considered antagonists. The nature of this antagonism is not entirely clear, since high-affinity hnRNP binding sites do not often overlap with SR protein binding sites in exons. A potential mechanism involves cooperative binding of hnRNP oligomers that extend along the transcript to prevent SR proteins from binding to pre-mRNA [24]. The best characterized hnRNPs involved in splicing regulation are the negative regulators hnRNP A/B and the PPT binding protein PTB, also known as hnRNP I. The hnRNPA2/B1 factor is mainly a splicing inhibitor that interferes with the recognition of 5’ss and 3’ss, which often leads to the exclusion of alternative exons (the functions of hnRNP A/B are detailed in [27]). PTB binds to polypyrimidine tracts, like U2AF65 does, which promotes the binding of U2 sn- RNP to the 3′ss. This implies that PTB may interfere with functional recognition of 3’ss [28]. The mechanism and direction of action of proteins belonging to the hnRNP family depends on the location of their binding sites: when binding upstream or inside the cassette exon, they usually act as repressors; when binding downstream, they act as activators of AS [19, 29, 30].

Besides SR and hnRNP proteins, several tissuespecific RNA-binding splicing regulators have been characterized. These include neuron-specific factors NOVA [31], PTBP2 (nPTB, brPTB) [32] and SRRM4 (nSR100) [33], as well as tissue-specific factors such as proteins of the RBFOX family [34], MBNL [35, 36], CELF [37], QKI [38], and TIA [39, 40]. They can exert their action through both tissue-specific expression and binding to pre-mRNA motifs that are enriched in genes expressed in a particular cell type or tissue. Tissue-specific regulators of AS are most often studied in relation with pathologies (e.g., neurodegenerative diseases or muscular dystrophy) [41, 42, 43].

The presence of RNA polymerase II is required for recruitment and proper distribution of splicing factors to their binding sites. Accordingly, transcription and splicing mutually influence each other through spatial and kinetic mechanisms [44]. RNA polymerase II has a C-terminal heptad repeat domain (CTD) that is used as a landing pad for accessible factors, allowing their concentration to increase near splice sites [45, 46, 47, 48]. The rate of transcription elongation influences AS by determining how quickly splice sites become available for competitive binding with *trans*-acting factors, particularly due to the formation of secondary structure in pre-mRNA [49, 50, 51, 52, 53].

## REGULATION OF AS BY PRE-mRNA SECONDARY STRUCTURE

Although most RNA molecules in a cell are single- stranded, their parts can adopt double-helical conformations, from which the secondary structure is formed. The secondary structure of RNA can be highly stable both *in vitro *and *in vivo*, and changes in its constituent elements are a well-known mechanism for the regulation of many cellular processes, including splicing [54, 55, 56, 57, 58].

Complementary base pairings forming RNA secondary structure can be classified as local and longrange interactions [59]. The simplest type of local RNA secondary structure is a hairpin (also known as stem-loop). Because pre-mRNA folding occurs cotranscriptionally, most of the *in vivo *RNA structure is generated through local interactions [60, 61]. In contrast, long-range interactions are formed between complementary sites separated by large fragments (more than 100 nt) of the primary sequence [62]. Long-range interactions share some features with the tertiary structure, yet they still represent the secondary level of organization, i.e., they determine how the polynucleotide chain is folded due to base pairings [59].

## LOCAL STRUCTURES IN PRE-mRNA

**Fig. 1:**
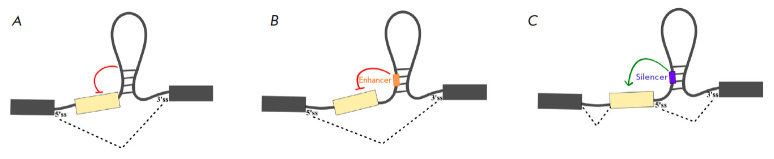
Blockage of *cis*-regulatory splicing elements by RNA structure. (*A*) Blockage of a splice site; (*B*) blockage of an intronic splicing enhancer; (*C*) blockage of an intronic splicing silencer. Red and green lines indicate the activating and inhibitory effects on splicing, respectively

Extensive experimental evidence exists for AS regulation by local pre-mRNA structure, for example, by preventing spliceosome recognition of the 5’ss, 3’ss, or BPS sequence elements [63]. The simplest mechanism of AS regulation by local secondary structure is the blockage of splice sites (*Fig. 1A*) [64]. Thus, in the pre-mRNA of the human *tau *gene, a local secondary structure obstructs the 5’ss of exon 10, which prevents this exon from being included in the mature transcript [65]. The formation of a hairpin near the 5’ss splice can interfere with the interaction of pre-mRNA with the spliceosome, as it does in the case of exon 7 of the *SMN2 *gene, where such a hairpin interferes with the binding of the 5’-ss to U1 snRNP, thus reducing the level of exon inclusion [66].

The pre-mRNA of the fibronectin gene (*FN1*) is the most striking example of the influence of the hairpin structure on the function of a splicing enhancer (*Fig. 1B*). One of the exons of *FN1*, known as the EDA exon, is highly structured and forms seven hairpins. The enhancer is located in the terminal loop of hairpin V and is recognized by *trans*-acting factors such as SRSF1. A change in the enhancer localization from a loop to a stem reduces its regulatory ability [67]. A similar mechanism of AS regulation involving an intronic splicing silencer is observed in the pre-mRNA of the human immunodeficiency virus (*Fig. 1C*) [68].

A non-canonical type of local secondary structure that influences AS is G-quadruplex (GQ). In a G-quadruplex, four guanosines interact with each other through Hoogsteen hydrogen bonds and their stacks form a four-stranded helix [69]. GQs act as* cis*-elements in AS regulation, usually reside in intronic regions, and promote exon inclusion. For example, disruption of the ability to form GQ significantly reduces the inclusion of exon 8 in the *CD44 *gene [70]. Several splicing regulators such as hnRNP H, hnRNP F, SRSF1, SRSF9, hnRNP U, and U2AF65 can interact with GQ [71, 72, 73]. The formation of GQ in the pre-mRNA of the *TP53 *gene in intron 3 regulates the splicing of intron 2, thus changing the ratio between the active and inactive isoforms [74]; intron retention leads to the generation of an inactive form of the protein, Δ40p53 [75].

Local secondary structures in pre-mRNA can also act as targets of small molecules. For example, 22 isoforms are generated as a result of AS of the transcript of the human telomerase reverse transcriptase gene (*hTERT*), of which only the full-length mRNA is translated into an active protein with reverse transcriptase activity [71]. The use of the GQ stabilizer reduces the level of active telomerase by eliminating exons 7 and 8. This leads to the synthesis of a truncated inactive protein called hTERT-β. Riboswitches are another important class of local RNA structures that influence AS and are targets of small molecules [76].

## LONG-RANGE INTERACTIONS IN PRE-mRNA, RNA BRIDGES, AND LOOP-OUTS

Long-range interactions in pre-mRNAs have been documented in viruses such as the tobacco mosaic virus [77], human immunodeficiency virus [78], etc. [79, 80]. The most remarkable example in eukaryotes, the Drosophila *Dscam *gene, is discussed below; however, we note here that more and more data support the presence of long-range interactions in human pre-mRNAs and their impact on AS [81, 82, 83, 84, 85, 86].

Long-range interactions can regulate AS by various mechanisms. First, like local RNA structures, they can block *cis*-regulatory elements [87]. Second, long-range interactions can act as “RNA bridges” that bring *cis*regulatory elements closer together [34]. Third, longrange interactions can also move *cis*-regulatory elements away from each other. For instance, long-range interactions between neighboring introns can loop out an intermediate exon or a group of exons and promote their skipping. The example of long-range in-teractions in the Drosophila *CG33298 *and *Gug *genes, which function as RNA bridges and simultaneously block splice sites [87], demonstrates that these three mechanisms are not mutually exclusive.

**Fig. 2:**
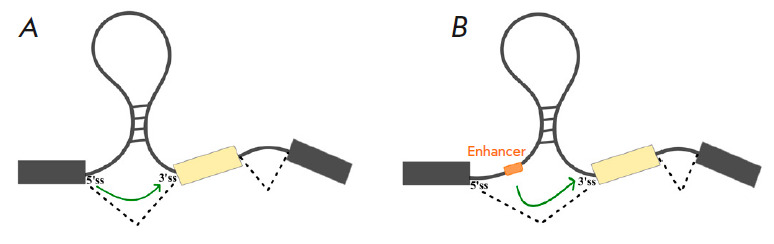
Spatial segregation of *cis*-regulatory splicing elements by RNA structure (RNA “bridges”). (*A*) Bringing splice sites closer together. (*B*) Bringing a splicing enhancer closer to the splice site

RNA bridges can bring *cis*-regulatory elements closer together in space without the participation of auxiliary proteins (*Fig. 2A*). For example, long-range interactions in the pre-mRNA of the mammalian *SF1* gene bring the strong 5’ss of exon 9 closer to the weak 3’ss of exon 10, and the destruction of the secondary structure leads to the activation of the stronger 3’ss located 21 nts downstream [62]. RNA bridges can also bring intronic *cis*-regulatory elements closer to splice sites (*Fig. 2B*). For successful assembly of the spliceosome and splicing of the *ENAH *gene, it is necessary that the binding site of the RBFOX2 factor be close in space to an alternative exon, which is achieved through the interaction of distant regions in the pre-mRNA forming an RNA bridge [34]. Many cases have been described in which *cis*-regulatory elements are located at a considerable distance from the regulated exon, such as in the Drosophila *14-3-3ζ* gene [88], as well as the human *ENAH *and *KIF21A* genes [34]. Genome-wide maps of RNA-protein interactions also show that the majority of binding sites are located much further than 1,000 nts from their potential target exons [89].

**Fig. 3:**
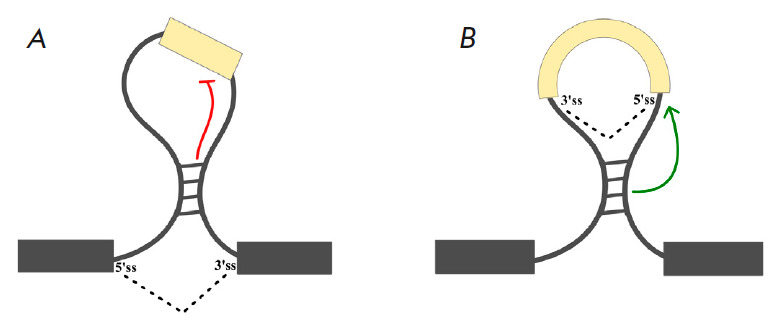
Spatial separation of *cis*-regulatory splicing elements by the RNA structure (loop-outs). (*A*) Looping out a region containing one or more exons. (*B*) Back-splicing in an intron leading to the formation of a circular RNA. Red and green lines indicate the activating and inhibitory effects on splicing, respectively

Looping out a part of pre-mRNA by secondary structure, on the one hand, can bring the flanking* cis*-regulatory elements closer together, and on the other hand, place the intervening sequence in a loop, which is believed to promote the exclusion of the looped-out region (*Fig. 3A*) [90]. For example, complementary interactions between the introns flanking an alternative exon tend to increase the frequency of its skipping [91]. The secondary structure in the Drosophila *Nmnat *gene loops approximately 350 nt and leads to the exclusion of exon 5 and the poly(A) signal from the pre-mRNA. In this case, the structure brings the distal acceptor splicing site closer to the donor site, thereby promoting the exclusion of skipped terminal exon [87]. Exon loop-outs are also characteristic of long-range interactions in other mammalian genes, for example, the *CASK *and* PHF20L1 *genes [92], the dystonin gene (*DST*), in which complementary regions presumably loop out a cluster of six exons [93], as well as the human telomerase gene (*hTERT*), in which long-range interactions between tandem repeats lead to skipping of two exons [94]. The example of the secondary structure in the pre-mRNA of proteolipid protein 1 (*PLP1*), the two alternative splice isoforms of which differ in the choice of an alternative 5’ss in the intron between exons 3 and 4, demonstrates that loop-outs not only of exons, but also of individual splice sites have a remarkable influence on splicing [95].

However, the most fascinating example of the influence of long-range interactions on AS is the Drosophila *Dscam *gene, in which complementary base pairings can occur at a distance of up to 12,000 nucleotides. A remarkable feature of the *Dscam *splicing mechanism is that complementary regions form a group of competing RNA structures that control the mutually exclusive choice of exons [96, 97]. The docker site located upstream of the exon 6 cluster can base-pair with only one of many selector sites located upstream of each of the alternative exons, thereby not only bringing together the distant 5’ss and 3’ss, but also looping out the intervening exons. The mutually exclusive mechanism of splicing is additionally controlled by hrp36, a factor that suppresses the ectopic inclusion of alternative exons promoted by SR proteins [98]. A similar mechanism was discovered in many other genes containing clusters of mutually exclusive exons (see review in [99]), e.g., example,* 14-3-3ζ *[100], *Mhc *[88], *srp*, *RIC-3*, *MRP1 *[101], *DNM1* [102], *TCF3*, *CD55 *[103], and *ATE1 *[52]. It has beensuggested that tandem duplications generating clusters of mutually exclusive exons inevitably lead to the formation of competing RNA structures and, consequently, to mutually exclusive AS [104].

However, placing a part of pre-mRNA in a loop does not prevent its binding to spliceosomal components and, on the contrary, can promote splicing. The example of circular RNAs shows that complementary interactions in introns, in particular the ones formed by Alu repeats, facilitate the so-called backsplicing that covalently links the 5’- and 3’-ends of RNA, resulting in the formation of circular transcripts (*Fig. 3D*) [105, 106]. In sum, it can be concluded that spatial segregation, spatial separation, and blockage of* cis*-regulatory elements by RNA structure are special cases of a more general molecular mechanism in which the splicing outcome is determined by transcript conformation, which, in turn, depends on longrange interactions in its secondary structure.

## COOPERATION AND COMPETITION OF RNA SECONDARY STRUCTURE AND RNA-PROTEIN INTERACTIONS

Pre-mRNA forms local secondary structure co-transcriptionally simultaneously interacting with RBPs [107]. RBPs contain well-defined RNA-binding domains (RBDs), such as RNA recognition domain (RRM), hnRNP K homology domain (KH), zinc fingers (ZF), etc., which interact with specific sequences and/ or structures in RNA [108]. Most RBDs recognize very short (3–7 nt) degenerate motifs, which are often organized in clusters. This increases the binding specificity of RBPs that contain multiple RBDs and also allows several RBPs to cooperate with each other [17]. For instance, high-affinity binding of the neuron-specific splicing factor NOVA is determined by the YCAY (Y = C/U) motif, which is usually found in clusters of several tetramers [109]. Some RBPs recognize spatially separated bipartite motifs that have a particular structural context [110]. However, RBPs recognizing similar motifs may have different binding profiles, and even high-affinity interactions may happen to be nonfunctional [111].

Multiple lines of evidence indicate that the most important factor influencing RBP binding is RNA structure [112]. RBP binding sites can be involved in various pre-mRNA structural elements [113]. It appears that ZF RBDs interact with RNA duplexes, as more than twenty ZF domain-containing RBPs selectively bind highly structured double-stranded microRNA precursors [108]. RBPs containing KH domains tend to prefer large hairpin loops. Given that most of these RBPs contain multiple RBDs, large hairpin loops allow simultaneous binding of multiple KH domains at once, as is the case with NOVA1 and PCBP2 [109, 114, 115, 116]. It can be assumed that the outcome of AS should depend on the balance between RNA–RNA and RNA–protein interactions, with the competition between them depending on the repertoire of the RBPs that are expressed in a given cell type [111]. Moreover, RBPs themselves often function combinatorially by binding to sites and structural elements on common mRNA targets [117].

**Fig. 4:**
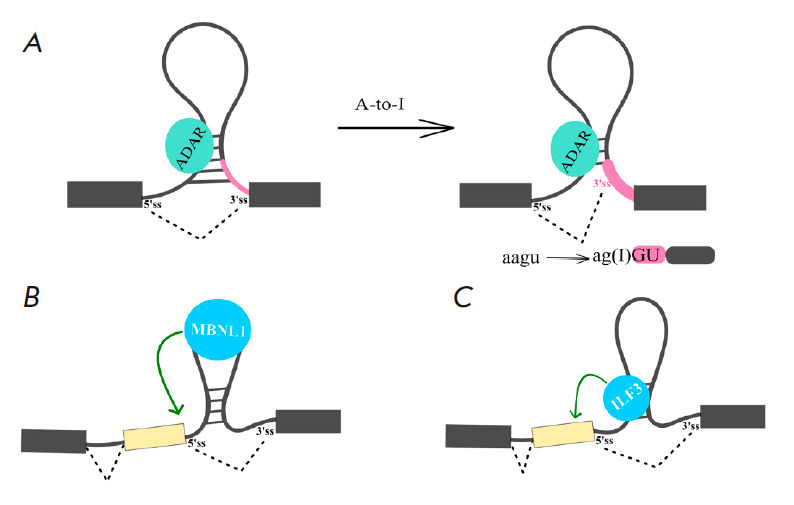
The combined effect of RNA secondary structure and RNA-protein interactions. (*A*) Creation of a splice site through RNA editing (A-to-I RNA editing). (*B*) Binding of an RNA-binding protein to a stem-loop structure. (*C*) Binding of an RNA-binding protein to a double-stranded region

Changes in RNA structure and the consequent changes in AS can occur due to interaction with other nucleic acids (e.g., with microRNA [118]), as well as a result of post-transcriptional modifications of the pre-mRNA primary sequence [119]. Thus, A-to-I editing performed by ADAR proteins regulates AS by changing the nucleotide sequence of the main splicing* cis*-elements (*Fig. 4A*) [120, 121, 122]. Additionally, ADAR2 can bind to the double-stranded RNA formed by the GA-rich sequence and polypyrimidine tract, thereby preventing U2AF65 recruitment [123]. Methylated N6- adenosine (m6A) and the associated proteins can regulate AS [119, 124]. For example, m6A modification can promote hnRNP C binding by altering the structure of the target RNA and exposing a single-stranded splice site. The same mechanism is also characteristic of hnRNP G [125].

RNA structure can obstruct *cis*-regulatory splice elements and RBP binding sites, but this is not the only way it can affect AS. Splicing of exon 5 of the human cardiac troponin T (*cTNT*) gene requires binding of the MBNL1 protein at the 3’ end of the upstream intron. MBNL1 binds to a part of the intron that forms a hairpin (*Fig. 4B*), whereas the splicing factor U2AF65 binds the same region when it is sin-gle-stranded. Stabilization of the local RNA structure in the form of a hairpin blocks U2AF65 binding, which prevents U2 snRNP recruitment and leads to exon skipping [126]. Another remarkable example is binding of hnRNP F to a pre-mRNA containing G-quadruplexes, which stimulates the inclusion of a cassette exon in the *CD44 *gene. Interestingly, another AS regulator, ESRP1, also stimulates the inclusion of the alternative exon in *CD44 *independently of hnRNP F by binding to a GU-rich motif partially overlapping with GQ. This suggests that *CD44 *pre-mRNA exists in equilibrium between linear and GQ forms, which allows to maintain the correct splice isoform ratio [70].

Regulation of AS can occur due to RBP-dependent stabilization or destabilization of RNA secondary structure [127]. For example, the ZFR (zinc-finger RNA-binding protein) and ILF3 proteins were shown to form heterodimeric duplexes with ILF2. The resulting complexes bind nonspecifically to doublestranded regions in the pre-mRNA, thereby affecting the accessibility of splice sites and the binding of* trans*-acting factors (*Fig. 4C*). The interaction of ILF3 and ZFR with RNA structure affects mutually exclusive choice of exons in the *ATE1 *gene. It was suggested that ZFR and ILF3 are involved in stabilizing RNA duplexes during mutually exclusive splicing, although the precise mechanism of their action remains unknown.

Some RBPs regulate AS by changing the premRNA tertiary structure. Unlike RNA bridges, in this case it is protein–protein rather than complementary interactions that induce pre-mRNA conformation that is necessary for AS. For example, homodimers of the hnRNPA1 protein interact with specific sites located in neighboring introns, bring them closer, and loop out the intervening exon, which leads to its skipping [90]. A similar mechanism is also characteristic of the hnRNP F/H proteins [128]. It was also shown that hnRNPA1 and hnRNP H can interact with each other and with other hnRNP family members [129]. The influence of the NOVA protein on splicing is also explained by spatial segregation of distant pre-mRNA regions, because its binding sites are often located at the beginning of the intron and near the BPS, which suggests that NOVA binds to two sites at the ends of the intron and forms a loop that brings the 5’ss and BPS closer together [130]. Homotypic and heterotypic interactions between RBPs, which bring remote regions of the pre-mRNA closer to each other, may be a widespread mechanism of AS regulation.

## CONCLUSION

AS regulation by RNA structure and AS regulation by RNA-binding proteins have been described previously as independent mechanisms. However, since binding of AS regulators may depend on RNA structure and, conversely, RNA structure formation may depend on interactions with regulators, multiple cross-talks between them exist. It is clear that the pre-mRNA structure is involved in the regulation of accessibility of splicing factor binding sites and contributes to the generation of conformations required for splicing through RNA bridges and loopouts. Protein factors can participate in modifying the pre-mRNA sequence, organizing its secondary and tertiary structure, thereby influencing the splicing outcome. Therefore, the local and long-range interactions in the structure of pre-mRNA and protein factors must be considered as inseparable parts of common regulatory cascades.

## Abbreviations

AS: alternative splicing
RBP: RNA-binding protein
snRNA: small nuclear RNA
snRNP: small nuclear ribonucleoprotein
5’ss: 5’ splice site
3’ss: 3’ splice site
PPT: polypyrimidine tract
BPS: branch point sequence
